# Hospital Meetings

**Published:** 1907-03-02

**Authors:** 


### HOSPITAL MEETINGS.

### ROYAL FREE HOSPITAL.

#### RETIREMENT OF MR. BURT.

The Right Hon. the Earl of Sandwich presided at the Annual Court of Governors of this hospital held in the hospital lecture-room on Monday last. The 78th report of the hospital was read and adopted. The report showed that the total income for 1906 had been £17,201 3s. 6d., and the total expenditure of £15,864 10s. 6d., an increase over that of 1905 accounted for by the largely increased number of patients treated. During the year the hospital treated 47,039 cases, of whom 2,474 were in-patients, while the number of attendances in the out-patients and casualty department totalled 110,353. The maternity department, which in its first year (1903) treated 132 cases, has fully justified its establishment, 417 cases having been treated during the year, but it has very materially increased the expenses. Various structural improvements—already detailed in our columns—have been made during the past year, but further accommodation is necessary. The Ladies' Association has done good work during the year and has decided to maintain a bed in Boys' ward. The chairman and other speakers emphasised the necessity for further improvements in the out-patients' department and for completing the work of structural improvements which had been commenced. The great need of the hospital was increased permanent support. Mr. Blackman, in moving the adoption of the report, quoted from the article we published last week on the hospital, and stated that close inquiry had been made into the circumstances of the in-patients; out of 334 cases so investigated, only four had been found in which there could be any doubt as to their suitability for admission. Mrs. Scharlieb strongly urged upon the Committee the desirability of providing new operating theatres.

#### A Tribute to Mr. Charles Burt.

It was moved by the Chairman, seconded by Sir Edwin Durning Lawrence, Bart., and unanimously resolved :—

"That the Governors desire to place upon record their high appreciation of the very valuable services which Mr. Charles Burt has for many years past so freely rendered to the hospital and the regret with which they have received his resignation of the office of treasurer. Mr. Burt joined the Committee of Management in the year 1889, and for ten years from 1895 to 1905 discharged the onerous duties of Chairman of the Weekly Board, and since 1901 he has held the office of treasurer.

"During the eighteen years that he has thus been associated with the management of the hospital he has at all times manifested the deepest interest in all that concerned its welfare. The many structural and other improvements which have been carried out during that period have been very largely due to his initiative and energy. Mr. Burt had also been a generous contributor to the hospital and on all occasions when it has been necessary to make special efforts to raise funds he has by his influence and energy rendered most valuable service to the Institution. The Governors rejoice to know that Mr. Burt will still retain his seat on the Committee of Management and Weekly Board, and while most heartily thanking him for all his past services they express the hope that he will long be spared to continue his beneficent labours on behalf of the Royal Free Hospital."

The Committee of Management was re-elected, Mr. Gamlen being appointed Treasurer. Votes of thanks to the Ladies' Association, to the Staff, and to the Chairman concluded the proceedings.

### CITY OF LONDON LYING-IN HOSPITAL.

The annual meeting took place on Wednesday, February 20, Mr. G. Berry in the chair. During the year the number of patients amounted to 3,425, exceeding that of any previous year in the history of the hospital; of these 639 were in-patients and 2,786 were delivered at their own houses. It was now the fourth year of the occupation of the temporary premises in Old Street, and in spite of the adverse conditions under which the work was carried on there had only been two maternal deaths, one being a case of embolism and the other of uraemic convulsions. Forty-two midwives and 113 monthly nurses had been trained during the year. The committee reported that Her Royal Highness the Princess of Wales had become Patroness of the hospital, and that the hospital had received a grant of £1,000 from King Edward's Hospital Fund, £220 from the Metropolitan Hospital Sunday Fund, and £35 10s. from the Hospital Saturday Fund. But they had already paid £30,048 on account of the new buildings, and had been obliged therefore to borrow from their bankers the sum of £24,500. They had lately received a deputation from a large London hospital asking them to receive medical students at the hospital, and the matter would have their full attention. Votes of thanks to the medical and nursing staff concluded the meeting.

### QUEEN CHARLOTTE'S.

The annual meeting was held on Monday last, Sir Samuel Scott, Bart., M.P., presiding. The report stated that 1,704 patients had been received into the hospital during the past year and 1,886 had been attended and nursed in their own homes. To the Convalescent Home 138 patients had been admitted. The ordinary expenditure had amounted to £6,331, while the ordinary income was £5,365, including grants of £500 from King Edward's Hospital Fund, £468 from the Hospital Sunday Fund, and £144 from the Hospital Saturday Fund. The arrangements for the enlargement of the Nurses' Home were progressing satisfactorily, and it was hoped to commence building in the spring. Mr. and Mrs. Bischoffsheim had given a munificent donation of £2,500 towards the cost of this enlargement, but upwards of £6,000 was still required. A large number of midwives and monthly nurses had been trained during the year, and 107 had passed the examination of the Central Midwives Board. In addition 24 students and 35 qualified practitioners had attended the practice of the hospital. The committee is most anxious to carry out this work without trenching on the small amount of capital the hospital possesses, and very earnestly appeals for the amount required to accomplish this object.

### THE LONDON HOMŒOPATHIC HOSPITAL.

The fifty-seventh annual meeting was held on Tuesday at the Hospital, the Right Hon. the Earl Cawdor, Treasurer of the hospital, presiding. The report stated that during the past year the work of the hospital had been carried on successfully. There had been an increase in both in-patients and out-patients, 1,183 in-patients having been treated and 25,626 out-patients, a record number of both since the hospital started in 1849. The financial year closed with a deficit of £402 on the year's working, as compared with £425 a year ago. The receipts include £1,000 from "An Opponent of Vivisection." The great effort made in 1904-5 had succeeded in getting the hospital out of debt to its capital funds by paying back the loan of £12,000. The income shows a steady increase. Strenuous efforts are to be made during the present year to raise the sum of £16,275 to complete the £30,000 required to extend the hospital building on its own freehold ground. The reasons for extension are: more ordinary ward accommodation is required; a children's observation ward is required to enable children suspected of infectious disease to be isolated for observation before admittance to the general ward; more out-patient accommodation is required. The Board contemplates wards for middle-class contributing patients, which are very much needed; also more domestic accommodation is urgently required. Sir Henry Tyler promises £10,000, and Lord Dysart £2,000 on condition that the £16,000 still required is promised or paid by December 31 next. The usual votes of thanks were passed to the officers and the medical staff.

The "Rapco" London view picture postcards, issued by the Regal Art Publishing Company, 9 Long Lane, are artistic and really excellently printed reproductions of well-known City views. The cards are entirely of British manufacture, and will appeal to collectors by their beauty of design and harmony of colour.

